# Effects of Cold and Vibration on Venipuncture Pain Management in Children Aged 2-7 Years Old: Randomized Controlled Trial

**DOI:** 10.2196/67918

**Published:** 2025-08-25

**Authors:** Zi-yun Zhou, Long-yi Hu, Ming-li Wang, Si-qi Li, Le-shan Zhou

**Affiliations:** 1 Hunan Provincial Comprehensive Childcare Service Center, Changsha, China; 2 Department of Clinical Nursing, Xiangya School of Nursing, Central South University, 172 Tong Zi Po Road, Yuelu District, Hunan, Changsha, China, 86 18975186606; 3 Teaching and Research Section of Clinical Nursing, Xiangya Hospital Central South University, Changsha, China

**Keywords:** external cold, pediatric nursing, children, childhood, venipuncture, phlebotomy, stress, procedural pain, pediatrics, toddler, cold, vibration, nonpharmacological, Buzzy device, Buzzy, Face, Legs, Activity, Cry, Consolability, Pain Behavior Scale, randomized, controlled trial, randomized controlled trial, RCT

## Abstract

**Background:**

The pain resulting from venipuncture is one of the primary sources of stress during childhood and can have adverse effects on children. The Buzzy device can play an important role in alleviating the pain of venipuncture in children.

**Objective:**

The aim of this study is to investigate the independent and combined effects of cold and vibration in reducing venipuncture pain and resistance behavior in children aged 2-7 years.

**Methods:**

A total of 130 children who underwent venipuncture were randomly assigned to 4 groups: 32 in the control group, 30 in the external cold group, 37 in the vibration group, and 31 in the cold combined with vibration group. The cold, vibration, and cold combined with vibration interventions used the Buzzy device. Nurses used the Face, Legs, Activity, Cry, Consolability Pain Behavior Scale and the Children’s Behavior Scale to assess pain and resistance behaviors before and during the venipuncture.

**Results:**

Compared with the control group, the cold group, vibration group, and cold combined with vibration group experienced milder pain and demonstrated weaker resistance behavior (*P*<.05). Pairwise comparison results showed that the combination group was superior to the vibration group (*P*<.05).

**Conclusions:**

External cold and vibration are effective in reducing procedural pain and improving compliance in children aged 2-7 years undergoing venipuncture. The cold combined with vibration has a synergistic effect, and the effect is better than vibration only.

## Introduction

### Background

Venipuncture is a common medical procedure, with moderate to severe pain stimulation [1]. Needle pricks are a significant part of early pain experiences [2]. Needle-related procedural pain is one of the primary sources of stress in childhood, causing fear in children [3]. Early pain experiences can affect physiological indicators and hormonal changes in children, potentially leading to reduced immunity and disrupted pain thresholds in the long term, which is detrimental to the development of the nervous system [4]. Furthermore, Cuenca-Martínez et al [5] suggest that children are more likely to develop a bias in pain memory toward acute pain. Therefore, improper pain management can lead to negative pain memories and needle phobia in children, which may not only increase pain but also trigger it [6]. However, many children do not receive adequate pain prevention and management during venipuncture [7].

The Buzzy device is a widely used nonpharmacological method for alleviating venipuncture pain in children. It is a bee-shaped tool that combines ice pack cooling and vibration. The device includes a vibration-generating engine powered by batteries, with a detachable ice pack as its wings. Compared to other nonpharmacological methods like virtual reality [8] and distraction interventions [9], it is simple to operate: you only need to place it approximately 5 centimeters above the puncture site and activate the device a few minutes before venipuncture. The Buzzy device’s soothing effect combines cold and vibration, two common and effective methods for reducing pain in children [10]. The individual use of cold or vibration has been well-validated in pediatric pain management [11]. Among nonpharmacologic strategies, the use of cold and vibration is a common and effective method for pain reduction in children [12]. Cold packs work by applying cold to a specific body area, which slows or inhibits pain transmission via peripheral nerves and induces vasoconstriction, thus raising the pain threshold [13]. Vibration stimulates tactile receptors in the skin, which localizes the sensation of pain and, when applied repetitively, reduces its intensity. This vibratory stimulation activates pain-blocking mechanisms and increases the release of β-endorphins, which raise the pain threshold and diminish the perception of pain [14,15]. The Buzzy device has been widely used to reduce pain during venipuncture in children [16].

However, researchers found that the combined use of cold and vibration with the Buzzy device did not seem as effective as imagined. Semerci et al [17] discovered that in school-aged children, using cold spray alone was more effective in relieving pain compared to the combined use of cold and vibration with the Buzzy device. Similarly, Ueki et al [18] found that for children under 6 years old, there was no difference in pain relief whether or not the vibration feature of the Buzzy device was activated when using an ice pack. Moreover, the pain relief effect of the Buzzy device might be due to the distraction caused by its bee-like appearance, which diminishes in older children [19]. Therefore, it seems there is a debate on whether the pain relief effect of the Buzzy device in children is due to the cold, the vibration, or the combination of both; current studies compare the Buzzy device with other pain management methods (such as cold spray) while neglecting the effects of the device itself. Compared to other nonpharmacological approaches, the Buzzy device is more cost-effective and easier to operate. If we can demonstrate that either cold application or vibration alone is effective, the Buzzy device could still be a viable option for pain relief—even if cold spray proves more effective.

According to a paper titled “A practical guide to acute pain management in children” [20], most pain assessment tools define age 7 as the threshold at which children can accurately self-report. Furthermore, Hedén et al [21] believe that children over the age of 7 are better able to distinguish between fear and pain. Younger children may develop a psychological and behavioral resistance to injections due to fear, increasing the risk of unsafe venipuncture [22]. For children under 2 years old, incomplete skin barrier function and thermoregulatory capabilities make them susceptible to skin damage, so cold is not recommended [23]. Therefore, this study selected children aged 2-7 as subjects to explore the independent effects and interactions of cold and vibration during the venipuncture process using the Buzzy device. The aim is to reduce pain and resistance behavior during venipuncture, enhance children’s adherence to treatments, and ultimately improve pain management in children.

### Objective

This study aims to investigate the independent and combined effects of cold and vibration in reducing venipuncture pain in children aged 2-7 years. The research hypothesis is that there are independent and interactive effects between vibration and external cold in solving procedural pain in children.

## Methods

### Participants

A randomized controlled trial was conducted to select children aged 2-7 years requiring venipuncture between May 2023 and November 2023 in the Department of Pediatrics of a tertiary hospital in Changsha City, Hunan Province, including the pediatric emergency department and pediatric ward, and the main reason for venipuncture was blood sampling, requiring peripheral intravenous access. This trial was reported using CONSORT (Consolidated Standards of Reporting Trials) guidelines. Informed consent was obtained from parents, and verbal assent was acquired from the children prior to their participation.

The inclusion criteria were (1) no history of cognitive or neurodevelopmental delay and (2) informed consent/assent obtained from both the children and their parents. The exclusion criteria were as follows: (1) difficulty in cooperation (ie, the child’s strong resistance to venipuncture, continuous struggle throughout the entire venipuncture process, and inability to cooperate with the use of the Buzzy device); (2) use of sedative drugs or treatments prior to venipuncture; (3) broken skin at the needle puncture site; (4) extreme sensitivity to cold (Raynaud’s syndrome or sickle cell disease); (5) experience with external cold or vibration within the past week; and (6) withdrawal of consent during venipuncture process.

While the child was waiting for the venipuncture, the researchers explained the purpose and methods of the study to the parents and obtained written consent from them. In addition, the researchers would explain the study to the child in simple and understandable language. If the child refused to participate, we would exclude the child from the study even if the parents agreed.

### Sample Size Calculation

The efficacy analysis of a 2-factor, 2-level ANOVA was performed using G*Power with settings of 1-β=.80, effect size=0.40, α=.05 according to the preexperimental results, a degree of freedom of 3, and 4 groups. Considering a 15% sample attrition rate, it was determined that each group should include at least 21 participants, requiring a total of 84 participants across the 4 groups.

A total of 145 children, aged 2-7 years and meeting the inclusion and exclusion criteria, were enrolled in the study. Participants were randomized into 4 treatment groups using IBM SPSS software (version 24.0; IBM Corp) based on the random number table method. Of the original cohort, 15 participants withdrew their consent by refusing to use the Buzzy tool during the venipuncture phase, leaving 130 participants for the final analysis.

### Ethical Considerations

The study was approved by the Ethics Review Committee of Xiangya School of Nursing, Central South University (E2023149) on June 23, 2023, and registered with the Clinical Trials Register (ChiCTR2400079536) on January 5, 2024. All participants were informed of the study’s purpose and the venipuncture process. Informed consent was obtained from parents, and verbal assent was acquired from the children prior to their participation. Participants did not receive any compensation. To protect the privacy of participants, we numbered them and did not retain any identifying information.

### Setting

Before starting the venipuncture, the researcher introduced the study to the children and their families. Due to the characteristics of the Buzzy device and the principle of informed consent, it was not possible to blind the children and their families. The nurses performing the venipuncture had all been working in pediatrics for over 5 years, held the title of “nurse-in-charge,” and had all undergone standardized pediatric venipuncture training, obtaining the necessary qualifications for conducting venipuncture.

**Figure 1.**
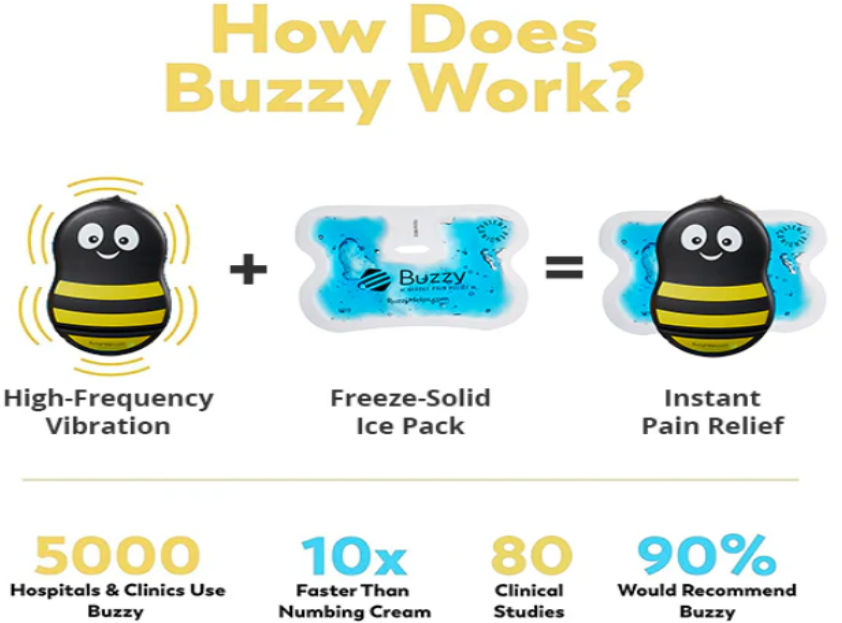
Buzzy device (image by MMJ Labs).

The Buzzy device (MMJ Labs) is a bee-shaped tool, measuring 8 × 5 × 2.5 cm, that integrates both vibration (the body of the bee) and cold (a removable ice pack). The ice pack is stored in the freezer and inserted into the device before the venipuncture. After use, it is returned to the freezer to maintain its cooling effect (Figure 1).

### Preparation for Venipuncture

The pediatric outpatient emergency injection room is designed to accommodate the needs of children, featuring decorations such as cartoon characters and warm colors. The room maintains appropriate levels of natural light, temperature, and humidity. Nurses ensure they are properly prepared, organize the necessary injection supplies and emergency equipment, and perform double-checks on medications to ensure accuracy.

### Venipuncture

For the control group, routine nursing care was implemented. The children’s and their families’ basic information was verified, and the purpose of the venipuncture was explained. According to the recommended usage of the Buzzy device [24], 1 minute before the injection, the Buzzy device was placed 5 centimeters above the puncture site before tightening the tourniquet. The cold pack and the vibration switch were not activated. An appropriate injection site and vessel were chosen, the area was disinfected twice, and the needle was positioned at a 15‐30° angle with the skin, bevel up, and advanced slightly after seeing blood return. Blood return was checked before advancing the needle slightly for fixation. The needle was secured and the Buzzy device was removed after securing the pillow. During the injection, the child was encouraged, engaged in conversation, and provided with timely feedback and praise. Postinjection, instructions on precautions were given, and the child was monitored for any discomfort or adverse reactions.

For the observation group, the other conditions were the same as the control group, but for one group, the vibration switch was activated, for another, an ice pack was applied for cold therapy, and the final group received both vibration and cold therapy.

### Data Collection

Before puncture (denoted as T_0_, no venipuncture or movement within the injection room) and during the puncture process (denoted as T_1_, from the application of the tourniquet to confirmation of successful puncture and fixation), two time periods were observed. Based on the scale, one research team member assessed the patient’s resistance behaviors while another evaluated the level of pain and recorded it.

### General Information

The child-specific information collected included gender, age, height, weight, punctured vein, whether it is the first intravenous injection, the time of the most recent injection, how many puncture attempts were needed for success, any hospitalization experience, presence of needle phobia, type of disease, and typical sensitivity to pain. This information was provided by the parents.

### Children’s Pain (Primary Outcome Measure)

The Face, Legs, Activity, Cry, Consolability (FLACC) Pain Behavior Scale is used to assess pain in children aged 2 months to 7 years or in individuals unable to communicate their pain. Each category is scored from 0 to 2, with a total score of 0 to 10. A score of 0 indicates no pain; 1‐3 indicates mild pain; 4‐6 indicates moderate pain; and 7‐10 indicates severe pain. Liu et al [25] translated it into Chinese, and it has demonstrated reliability and validity, with a Cronbach α of 0.88.

### Behavioral State

A compliance and resistance behavior assessment was conducted for each child. This involved evaluating the child’s behavior during the injection, including crying, screaming, agitation, struggling, and avoidance. A scoring system of 0-3 was used, with 5 items in total and a maximum score of 15. The higher the score, the poorer the compliance and the stronger the resistance behavior [22].

### Statistical Analysis

Statistical analysis was conducted using SPSS software (version 24.0; IBM Corp). Measurement data were described as mean (SD), and ANOVA was used for comparisons between groups. Categorical data were described using frequency and composition ratios, and comparisons were made using the chi-square test and Fisher exact test. The significance level was set at 0.05. For measurement data that did not follow a normal distribution or exhibited heterogeneity of variance, the median (percentile) was used for description, and intergroup differences were analyzed using nonparametric rank-sum tests (Kruskal-Wallis rank-sum test). In cases of homogeneity of variance, post hoc multiple comparisons were performed using the Bonferroni test.

## Results

### Demographic Information

**Figure 2.**
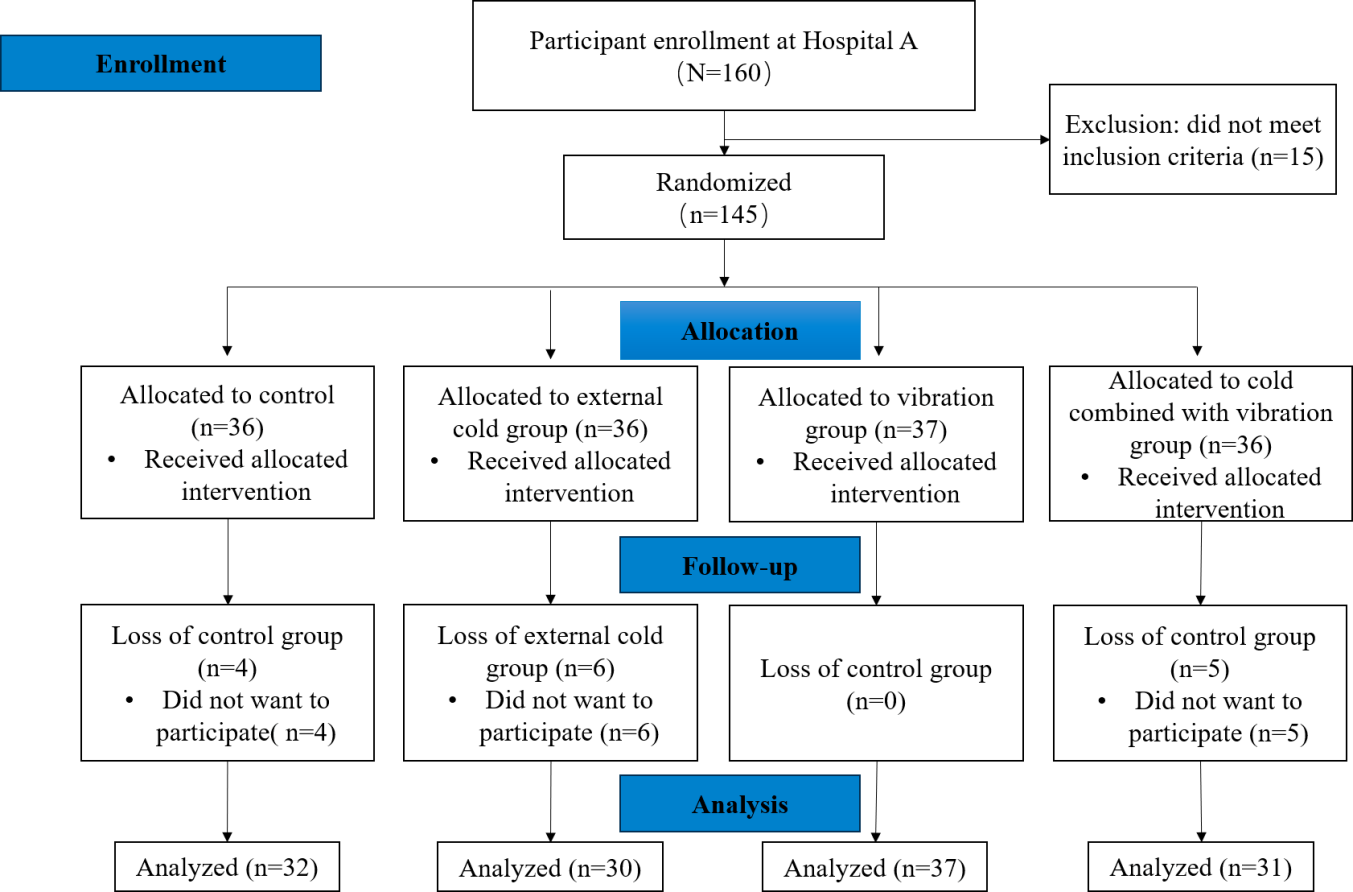
Flow diagram.

A total of 145 children were enrolled in the study and randomized into 4 treatment groups. Of these, 15 participants withdrew their consent, leaving 130 participants for the final analysis. The flowchart of participant allocation is shown in Figure 2.

A total of 130 sets of data on children aged 2-7 years were collected, including 32 in the control group, 30 in the external cold group, 37 in the vibration group, and 31 in the combined cold and vibration group. The results showed that the average age of patients in the control group, external cold group, vibration group, and combined cold and vibration group was 3.97, 4.03, 4.00, and 3.77 years, respectively. There were no statistically significant differences in demographic variables between control, vibration, cold, and combined treatment groups (*P*>.05), indicating a general comparability, as shown in Table 1.

**Table 1.** Demographic information (N=130).

Items	Control (n=32)	External cold (n=30)	Vibration (n=37)	Cold combined with vibration (n=31)	*F* test (*df*=3) or chi-square (*df*=3)	*P* value
Gender, n (%)					0.978^a^	.81
Male	16 (50)	16 (53.3)	19 (51.4)	19 (61.3)		
Female	16 (50)	14 (46.7)	18 (48.6)	12 (38.7)		
Age (years), mean (SD)	3.97 (1.69)	4.03 (1.71)	4.00 (1.76)	3.77 (1.63)	0.146^b^	.93
Height (cm), mean (SD)	105.25 (11.46)	107.27 (12.04)	104.49 (11.50)	104.63 (11.97)	0.373^b^	.77
Weight (kg), mean (SD)	16.68 (4.27)	17.38 (3.62)	17.49 (4.81)	16.52 (3.91)	0.438^b^	.73
BMI, mean (SD)	14.94 (1.69)	15.20 (1.79)	15.95 (2.24)	15.02 (1.35)	2.281^b^	.08
Last injection time, n (%)					0.659^a^	.88
≤7 days	26 (81.2)	22 (73.3)	28 (75.7)	23 (74.2)		
>7 days	6 (18.8)	8 (26.7)	9 (24.3)	8 (25.8)		
Puncture success, n (%)					1.734^a^	.84
Once	31 (96.9)	29 (96.7)	35 (94.6)	31 (100)		
Twice	1 (3.1)	1 (3.3)	2 (5.4)	0 (0)		
Vessel site, n (%)					6.568^a^	.09
Back of the hand	21 (65.6)	25 (83.3)	27 (73)	28 (90.3)		
Other	11 (34.4)	5 (16.7)	10 (27)	3 (9.7)		
Disease, n (%)					7.706^a^	.26
Respiratory system	19 (59.4)	22 (73.3)	20 (54.1)	23 (74.2)		
Blood system	8 (25)	3 (10)	6 (16.2)	5 (16.1)		
Other	5 (15.6)	5 (16.7)	11 (29.7)	3 (9.7)		
Experience of hospitalization, n (%)					1.454^a^	.69
No	13 (40.6)	14 (46.7)	19 (51.4)	17 (54.8)		
Yes	19 (59.4)	16 (53.3)	18 (48.6)	14 (45.2)		
Needle phobia, n (%)					1.436^a^	.70
No	14 (43.8)	14 (46.7)	18 (48.6)	18 (58.1)		
Yes	18 (56.2)	16 (53.3)	19 (51.4)	13 (41.9)		
Sensitivity to pain, n (%)					3.019^a^	.82
Not	2 (6.3)	2 (6.7)	5 (13.5)	3 (9.7)		
General	21 (65.6)	19 (63.3)	23 (62.2)	16 (51.6)		
Very	9 (28.1)	9 (30)	9 (24.3)	12 (38.7)		

a Chi-square.

b *F* test.

### Impact of External Cold and Vibration on Pain and Resistance Behavior

After comparing pain (*P*=.26) and compliance resistance behavior scores (*P*=.31) among the groups of children at the T_0_ stage, the differences were not statistically significant. The differences at the T_1_ stage were statistically significant (*P*<.001). Pairwise comparison results showed that at T_1_, the pain scores of the control group were higher than those of the external cold, vibration, and combination group (*P*<.001), and the vibration group had higher pain scores than the combination group (*P*=.03). Furthermore, the mean pain scores from highest to lowest were as follows: control group (4.38, SD 2.25), vibration group (1.81, SD 1.4), external cold group (1.10, SD 1.77), and combination group (0.65, SD 1.20). At T_1_, the resistance behavior of the control group was higher than that of the external cold, vibration, and combination group (*P*<.001), and the vibration group exhibited higher resistance behavior than the combination group (*P*=.01), with the differences before and after decreasing in the following order: control group (mean 2.97, SD 2.39), vibration group (mean 1.08, SD 1.23), external cold group (mean 0.57, SD 1.89), and combination group (mean 0.26, SD 0.93) (Table 2).

**Table 2.** Comparison of pain and resistance behavior scores.

Items	Control (n=32)	External cold (n=30)	Vibration (n=37)	Cold combined with vibration (n=31)	*F* test (*df*=3) or *H* test	*P* value
FLACC^a^						
T_0_, mean (SD)	1.97 (2.25)	3.00 (2.36)	2.16 (2.48)	2.68 (1.88)	1.366^b^	.26
T_1_, mean (SD)	6.34 (2.96)^c^^,^^d^^,^^e^	4.10 (2.62)^f^	3.97 (2.98)^f^	3.32 (1.92)^f^	7.759^b^	<.001
△, mean (SD)	4.38 (2.25)^c^^,^^d^^,^^e^	1.10 (1.77)^f^	1.81 (1.41)^e^^,^^f^	0.65 (1.20)^d^^,^^f^	16.293^b^	<.001
Resistance behavior						
T_0_, median (IQR)	5 (5-6)	6 (5-7)	5 (5-7)	6 (5-6)	3.580^g^	.31
T_1_, mean (SD)	8.72 (3.22)^c^^,^^d^^,^^e^	6.67 (1.88)^f^	7.16 (2.18)^f^	6.19 (1.35)^f^	7.364^b^	<.001
△, mean (SD)	2.97 (2.39)^c^^,^^d^^,^^e^	0.57 (1.89)^f^	1.08 (1.23)^e^^,^^f^	0.26 (0.93)^d^^,^^f^	16.293^b^	<.001

a FLACC: Face, Legs, Activity, Cry, Consolability.

b *F* test.

c Different from the external cold group.

d Different from the vibration group.

e Different from the cold combined with vibration group.

f Different from the control group.

g Kruskal-Wallis test.

### The Independent and Combined Effects of External Cold and Vibration Intervention

**Figure 3.**
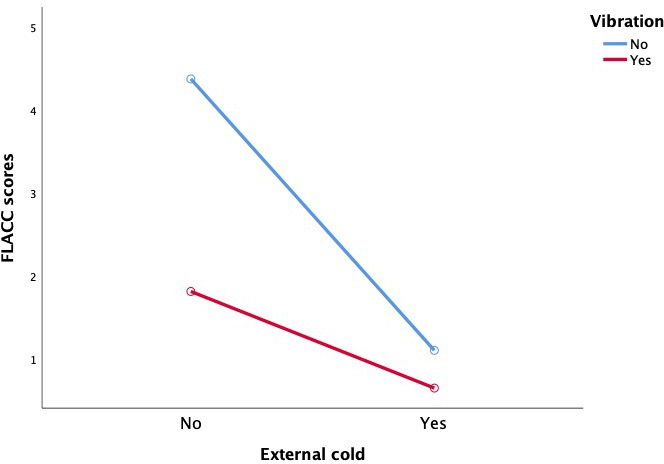
Comparison of mean FLACC score values resulting from cold and/or vibration intervention in children aged 2-7 years old. FLACC: Face, Legs, Activity, Cry, Consolability.

Both external cold and vibration had statistically significant main effects on reducing pain and compliance-resistance behaviors in children (*P*<.001), and there was a statistically significant interaction between external cold and vibration (FLACC scores: *F*=12.469, *P*=.001; resistance behavior: *F*=7.025, *P*=.009), as shown in Table 3 and Figures3  4.

**Table 3.** Analysis of group differences and interaction effects in pain and compliance-resistance behavior scores.

Intervention factor	Type III sum of squares	Mean square	*F* test (*df*=1)	*P* value
FLACC^a^				
External cold	159.196	159.196	55.262	<.001
Vibration	73.582	73.582	25.543	<.001
Interaction (cold × vibration)	35.920	35.920	12.469	.001
Resistance behavior				
External cold	83.970	83.970	29.306	<.001
Vibration	38.941	38.941	13.591	<.001
Interaction (cold × vibration)	20.130	20.130	7.025	.009

a FLACC: Face, Legs, Activity, Cry, Consolability.

**Figure 4.**
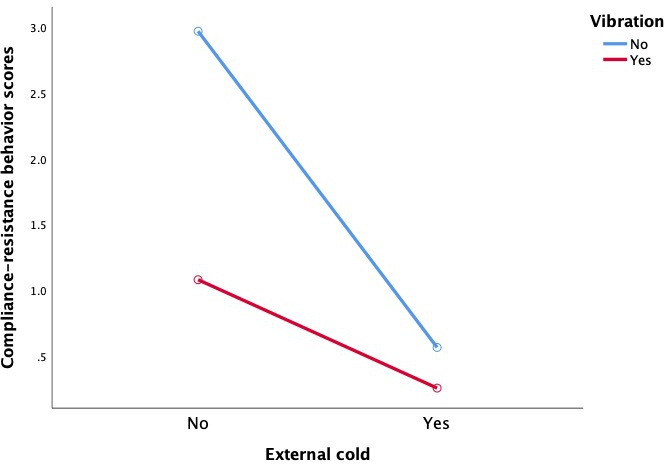
Comparison of the effect of cold and/or vibration intervention on compliance-resistance behaviors in children aged 2-7 years old.

## Discussion

### Main Finding

The main finding of this study is that cold and vibration methods can effectively reduce resistance behavior and increase compliance, while decreasing pain in children aged 2-7 years during venipuncture. The cold combined with vibration is superior to external cold or vibration alone.

### Impact of External Cold and Vibration on Pain and Resistance Behavior

The findings of this study are consistent with previous research [26-28]. Ballard et al [24] suggested the Buzzy device works through gate control theory. According to this theory, nonpainful stimuli (like touch, pressure, and temperature) carried by large nerve fibers can inhibit pain signals transmitted by smaller A-δ and C fibers. Applying external cold and vibration before venipuncture generates nonpainful sensory signals that activate inhibitory functions in neural transmission, causing the gate that regulates pain signals entering the brain to close, blocking pain signal transmission and thereby reducing pain [29]. The cold and vibration also trigger β-endorphin release, raising pain thresholds. Additionally, external cold causes vasoconstriction, increasing the pain threshold and reducing pain perception [12], while vibration stimulates skin tactile receptors, such as Pacinian and Meissner’s corpuscles, confining pain to a local area [30]. Furthermore, these stimuli distract children from pain, increasing their comfort [31].

Current research mostly focuses on the effect of reducing pain in children during venipuncture, neglecting the assessment of behavioral state effects [12,32]. Our research explores this. On the one hand, by effectively reducing venipuncture pain, the child’s resistance to the venipuncture is diminished; on the other hand, it serves as an effective distraction, lowering the child’s anticipatory anxiety about the venipuncture, thereby reducing irritability and restlessness [33]. Cold and vibration can decrease discomfort and uneasiness in children during venipuncture, helping them to overcome negative perceptions and responses, increasing compliance, and improving the success rate of venipuncture [34].

### The Independent and Combined Effects of External Cold and Vibration Intervention

The study results indicate that during venipuncture in children aged 2-7 years, cold and vibration interventions have both independent effects and interaction effects on reducing resistance behavior and pain. That is, the effects of cold or vibration can change with the level of either intervention, with a synergistic interaction between them, enhancing the effects when combined. The group receiving both external cold and vibration showed a better effect in reducing resistance behavior and alleviating venipuncture pain than the groups receiving only external cold or only vibration. This suggests that the cold combined with vibration is more beneficial, as demonstrated by Faghihian et al’s results [35], which indicated that combining external cold with vibration was more effective in alleviating anesthesia injection pain in children aged 6-12 compared to using only an ice bag to apply external cold. External cold and vibration can each play a role by blocking pain signal transmission and affecting neurotransmitter secretion mechanisms, respectively, reducing pain perception and increasing pain thresholds [36], thereby minimizing the children’s resistance behavior due to pain.

There was no difference in reduced pain results when using vibration with cold. Ueki et al [18] also found that when children’s pain was assessed by researchers, there was no significant difference in pain relief from vaccination when using only cold or a combination of both cold and vibration. However, they did not further explore the difference between using the Buzzy device and not. This “1+1<2” result may be related to the limited interception of pain signals by nonpain sensory signals provided by cold application and vibration. The specific reasons require further investigation. The individual use of cold and vibration is more convenient, and future studies could conduct a cost-effectiveness analysis of using the Buzzy device to apply cold, vibration, and cold combined with vibration.

### Clinical Implication

This study demonstrated the effectiveness of the Buzzy device for pain management in children aged 2‐7 years, providing a theoretical basis for clinical practice in pediatric pain management.

### Limitations

The participants in this study all came from the same hospital and only included children aged 2‐7 years, which limits its representativeness. Future studies could implement multicenter research and include all age groups. The effect of external cold in this experiment may vary with different seasons and temperatures, and this study did not compare the minimal clinically important difference, which could be further explored in future research.

### Conclusion

The application of cold and vibration methods has been shown to significantly mitigate resistance and enhance compliance, concurrently diminishing pain in children aged 2-7 years undergoing venipuncture. The combined effect of cold and vibration was superior to vibration alone, but no different from cold compress alone. Future research could focus on the cost-effectiveness analysis of using the Buzzy device for cold, vibration, and both interventions.

## Supplementary material

10.2196/67918 Checklist 1 CONSORT (Consolidated Standards of Reporting Trials) checklist.

## Abbreviations

CONSORT: Consolidated Standards of Reporting Trials
FLACC: Face, Legs, Activity, Cry, Consolability
